# Delayed Onset Neurological Deterioration due to a Spinal Epidural Hematoma after a Spine Fracture

**DOI:** 10.4184/asj.2007.1.2.98

**Published:** 2007-12-31

**Authors:** Jung Won Ha, Jin Oh Park, Eun Su Moon, Chong Hyuk Choi, Ju Young Kim, Hak Sun Kim, Jeong-Gil Lee

**Affiliations:** * Department of Orthopedic Surgery, National Health Insurance Corporation Ilsan Hospital, Koyang, Korea.; † Department of Orthopedic Surgery, Youngdong Severance Hospital, Yonsei University, Seoul, Korea.

**Keywords:** Spinal epidural hematoma, Spine fracture, Neurology

## Abstract

There are no reports of a 7-day delay in the onset of neurological deterioration because of a spinal epidural hematoma (SEH) after a spinal fracture. A hematoma was detected from the T12 to L2 area in a 36-year-old male patient with a T12 burst fracture. On the same day, the patient underwent *in situ* posterior pedicle instrumentation on T10-L3 with no additional laminectomy. On the seventh postoperative day, the patient suddenly developed weakness and sensory changes in both extremities, together with a sharp pain. A MRI showed that the hematoma had definitely increased in size. A partial laminectomy was performed 12 hours after the onset of symptoms. Two days after surgery, recovery of neurological function was noted. This case shows that spinal surgeons need to be aware of the possible occurrence of a delayed aggravated SEH and neurological deterioration after a spinal fracture.

## Introduction

A spinal epidural hematoma (SEH) is a rare entity that was first reported by Jackson in the 17th century^1^. An SEH occurs spontaneously in most cases. However, in some cases, an SEH occurs due to vascular abnormalities such as an arterio-venous malformation (AVM), a vertebral hemangioma, obstetrical birth trauma, and a spinal trauma^2^.

The frequency of an SEH related to spinal fractures has been reported to range from 0.5 to 7.5%^1^,^3^. Foo and Rossier^3^ reported that although an SEH caused by a spinal fracture is rare, it requires surgical decompression because an acute progressive neurological deficit appears in most cases. We encountered a patient who did not show any neurological deficit immediately after a spinal fracture. However, 7 days after the spinal fracture fixation, the patient suddenly developed delayed neurological deterioration on both lower extremities. This is the first report showing an increase in size of an epidural hematoma on a repeated MRI after the exacerbation of the neurological deficit.

## Case Report

**Fig. 1:**
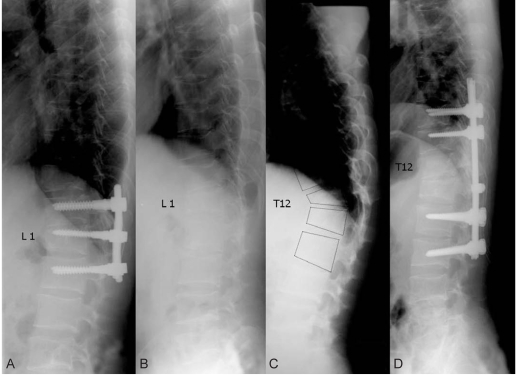
T-L spine lateral radiograph. He had been treated for an unstable L1 burst fracture using posterior instrumentation on the T12-L2 and anterior interbody fusion with autoiliac bone graft 3 years ago (**A**), and one year ago he had the posterior instrument removed (**B**). There was a burst fracture with distraction force on T12 (**C**), and he underwent in situ posterior pedicle instrumentation on the T10-L3 (**D**).

**Fig. 2:**
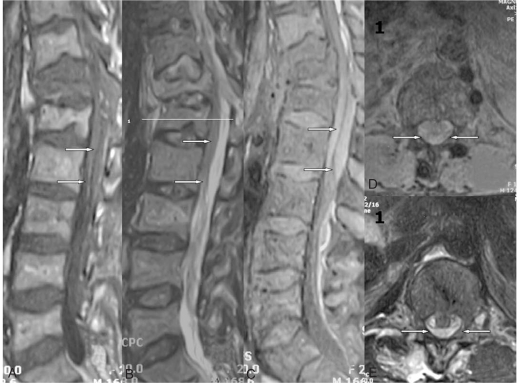
MRI findings on the initial trauma. Isotense on the T1-weighted image (**A**), hyperintense hematoma on the T2 sagittal and enhanced MRI (**B**, **C**), and hematoma compressing the cord on the T1 and T2 axial image (**D**, **E**).

A 36-year-old male patient was admitted to our hospital with paraparesis on both lower extremities that had developed suddenly 2 hours prior to admission. Seven days prior the patient had suffered back pain as a result of a direct fall on the lumbar region, and was diagnosed with a burst fracture with a distraction force on T12 (Fig. 1C). The patient underwent an MRI examination on the thoraco-lumbar area on the same day at another hospital (Fig. 1D). The MRI showed a collapse of the T12 vertebral body and T12-L1 inter-spinous widening. The lesion was isointense on T1-weighted images and hyperintense on T2-weighted images. A hematoma was detected from the T12 to L2 area. The hematoma displaced the spinal cord and cauda equina (Fig. 2). No neurological deficits were noted. On the same day, the patient underwent *in situ* posterior pedicle instrumentation on T10-L3 but a laminectomy was not performed. Non-steroidal anti-inflammatory drugs, which influence blood coagulation, were not administered after surgery. A postoperative neurological status was not detected. Three days after surgery, the patient was encouraged to ambulate, and could walk without difficulty.

Seven days after surgery, the patient suddenly developed weakness and sensory changes in both extremities together with a sharp pain. Consequently, the patient was transferred to the emergency room. In the initial neurological examination performed in the emergency room, the strength of the hip flexion on the right side was trace (1/5) and was zero below the knee (0/5). On the left side, the ankle dorsiflexion was poor (2/5), and was zero below the ankle (0/5). The sensory was decreased to below the L1 dermatome. The knee and ankle jerk were decreased, and the bulvocavenous reflex and anal tone were also decreased.

**Fig. 3:**
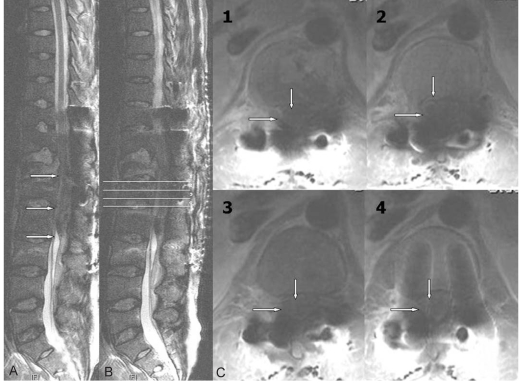
MRI with neurological deficit 7 days after the spinal trauma and fixation. Heterogeneous hypointense hematoma on the T2-weighted images was noted (**A**, **B**), and it was definitely increased on axial plane compared with initial axial images (**C**).

Medical records revealed that the patient had been treated in our hospital for an unstable L1 burst fracture 3 years prior using posterior instrumentation on T12-L2 and anterior interbody fusion with an autoiliac bone graft. One year ago, the patient had the posterior instrument removed (Fig. 1A and B). No bleeding tendency was noted in the medical history. A laboratory test showed that the blood profile *i.e.* the platelet count, prothrombin time, activated prothombin time, bleeding time, and coagulation time were within the normal ranges. An MRI performed 6 hours after the development of symptoms in the emergency room showed a heterogeneous isointense lesion on T1-weighted images, and a hematoma that was heterogeneous hypointense on T2-weighted images that had definitely increased in size, as detected on the sagittal plane and axial plane images (Fig. 3).

Twelve hours after the development of symptoms, a partial T12-L3 laminectomy was performed under general anesthesia. The hematoma filled the epidural space completely and ranged from the T12 to L2 level. A hematoma evacuation was performed, and no definite bleeding focus was detected. Two days after surgery, a recovery of neurological function was noted. One year after surgery, the hypoesthesia was below the right L5 dermatome, the ankle dorsiflexion on the right side was good (4/5) but was slightly lower than normal. However, the other neurological functions had returned to normal.

## Discussion

Since a spinal epidural hematoma was first reported in the 17th century, there have been more than 260 case reports^1^. Many cases have been spontaneous and most often there has been an association with coagulopathy or the use of anticoagulant therapy. However, rare cases of an SEH due to a spinal fracture, obstetrical birth trauma, lumbar puncture, post-surgical bleeding, epidural anesthesia, and missile injury have also been reported^2^,^3^. Of these cases, an SEH associated with a spinal fracture is rarer, and the incidence has been reported to range from 0.5% to 7.5%^3^,^4^.

The most common site of an SEH in males is the lower cervical and thoracolumbar spine, and the lower thoracic spine is the most common site of an SEH in females. The bleeding associated with an SEH is known to originate from the venus plexus in the epidural space. The causality has been reported to be an obstruction of the valveless epidural vein plexus in response to trauma or transmission of a suddenly increased intrathoracic or intraabdominal pressure^4^,^5^. In addition, epidural hemorrhage is frequently located dorsal to the spinal cord because of the tight fixation of the dura to the vertebral bodies^6^,^7^.

It has been reported that in most cases of an SEH accompanying a spinal trauma particularly fractures, acute sensory and motor deficits develop within minutes to hours, and within 3 days in rare cases. The SEH subsequently progresses to complete paralysis or the loss of urinary control or sphincter function^3^,^8^. Foo and Rossier^3^ reported that in a comparison of patients with accompanying spine fractures and those without fractures, the interval of the trauma and the onset of paresis was shorter in those patients with accompanying spine fractures.

In our case, MRI was performed immediately after the trauma, and the SEH was detected from the T12 to L2 level. However, a neurological deficit did not develop for 7 days. Although we are unable to explain the pathomechanism of the delayed onset of the neurological deterioration, compared with the MRI taken after the development of the neurological deficit, an increase in the size of the epidural hematoma was detected at the same level. Therefore, it is speculated that the result of such an increased hematoma may be the instability caused by the encouragement of early walking, and the increase may mediate the neurological deterioration. Uribe et al.^2^ reported that the incidence of delayed symptomatic SEH cases after spinal surgery increased significantly in patients with a prior surgical history. These investigators attributed this to the reduced elasticity of the scar tissues resulting in decreased blood absorption ability. A similar effect may be applied to our patient who had a prior burst fracture on the L1 vertebra.

This report is the first to describe the delayed onset of neurological deterioration due to an SEH after a spinal fracture. An increase in the hematoma volume, where the neurological symptoms might have developed after the spinal fracture (even after 7 days), was demonstrated on repeated MRI performed after the exacerbation of the neurological symptoms.
